# Supplementary material for: The Rad53CHK1/CHK2-Spt21NPAT and Tel1ATM axes couple glucose tolerance to histone dosage and subtelomeric silencing
Source: Nat Commun. 2020 Aug 19;11:4154. doi: 10.1038/s41467-020-17961-4 (PMC7438486; doi:10.1038/s41467-020-17961-4)
Supplement: Supplementary file 1 — Supplementary Information [file 41467_2020_17961_MOESM1_ESM.pdf]

The Rad53$^{CHK1/CHK2}$-Spt21^{NPAT} and Tel1^{ATM} axes couple glucose tolerance to histone dosage and subtelomeric silencing

Bruhn et al.

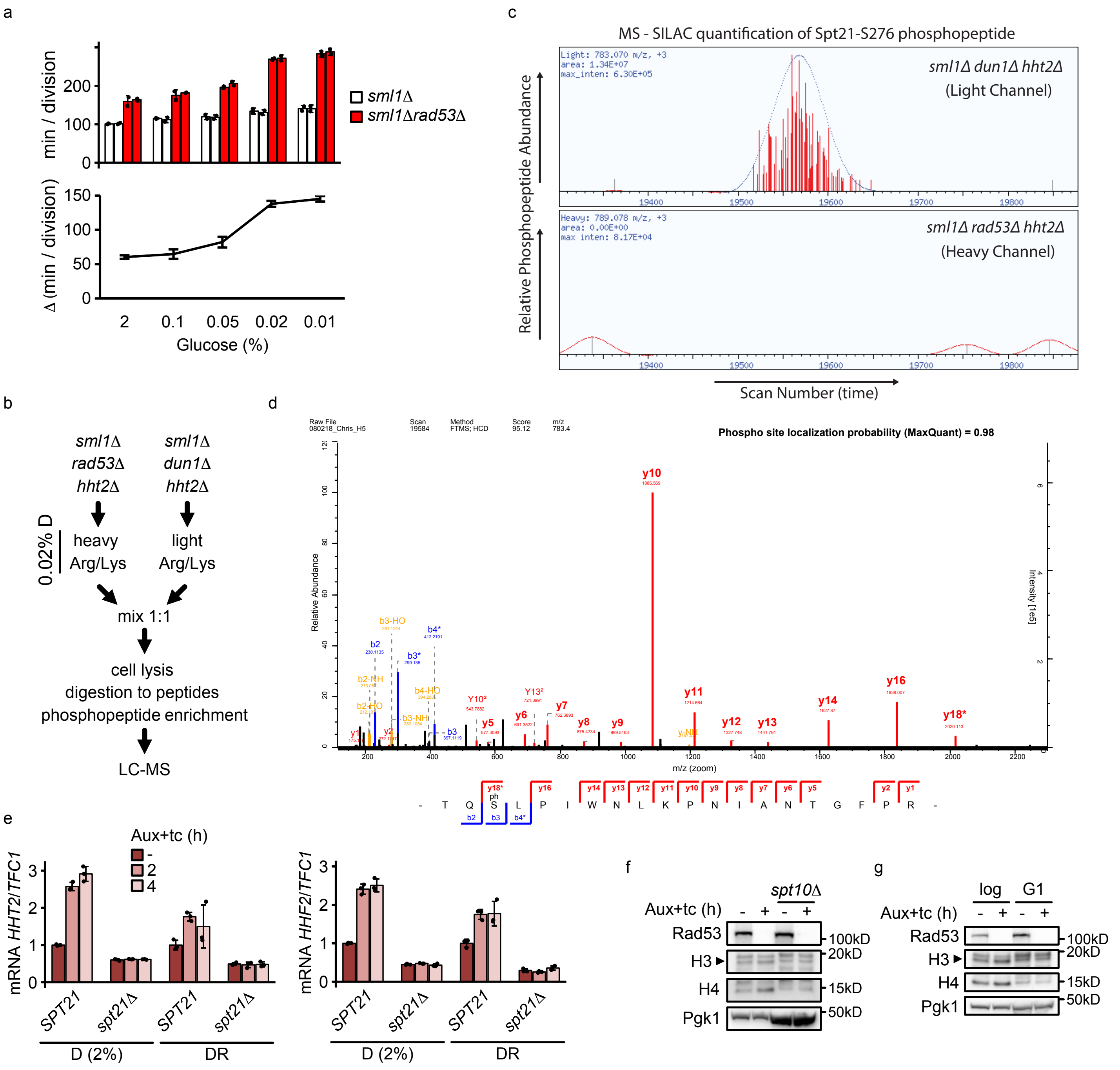

Supplementary Figure 1. Rad53 regulates Spt21 in low glucose

(a) Cells were adapted to the indicated glucose concentrations, and cultures were inoculated at low density. Cells were counted after 16 h and doubling times were calculated. N = 3 replicates of 2 independent clones.

(b) Workflow for SILAC-based quantification of Rad53-dependent phosphorylation events in glucose limiting growth conditions.

(c) SILAC quantification of the Spt21-S276 phosphopeptide. Quantification was performed using EXPRESS. Image captured from web based SORCERER viewer from Sagen.

(d) Sequest-based Peptide-Spectrum match (PSM) for the Spt21-S276 phosphopeptide. Image captured from web-based SORCERER viewer from Sagen.

(e) Rad53-AID cells with the indicated *SPT21* genotype were adapted to normal or low glucose and treated with Auxin and tetracycline as indicated to deplete Rad53. Samples were collected to quantify histone mRNAs by RT-qPCR. Data of Figure 1e are contained in this Figure. n = 3 independent replicate cultures.

(f) Rad53-AID cells with the indicated *SPT10* genotype were adapted to low glucose and treated as indicated with Auxin and tetracycline for 4h to deplete Rad53. Samples were collected to detect histones by Western blot analysis. Lysate corresponding to an equal amount of cells was loaded per lane. The difference in Pgk1 levels likely represents a regulation by Spt10.

(g) Rad53-AID cells were adapted to low glucose and arrested with α factor for 1h as indicated (G1). Rad53 depletion was induced by addition of Auxin and tetracycline, in the presence of α factor where indicated (G1). Samples were collected after 2h to detect Rad53 and histones by Western blot analysis.

Data are represented as mean, error bars indicate the standard deviation, D = glucose, DR = glucose restriction (0.02% in liquid media), Aux = Auxin, tc = tetracycline, MS = mass spectrometry, SILAC = Stable Isotope Labeling with Amino acids of Cells in culture.

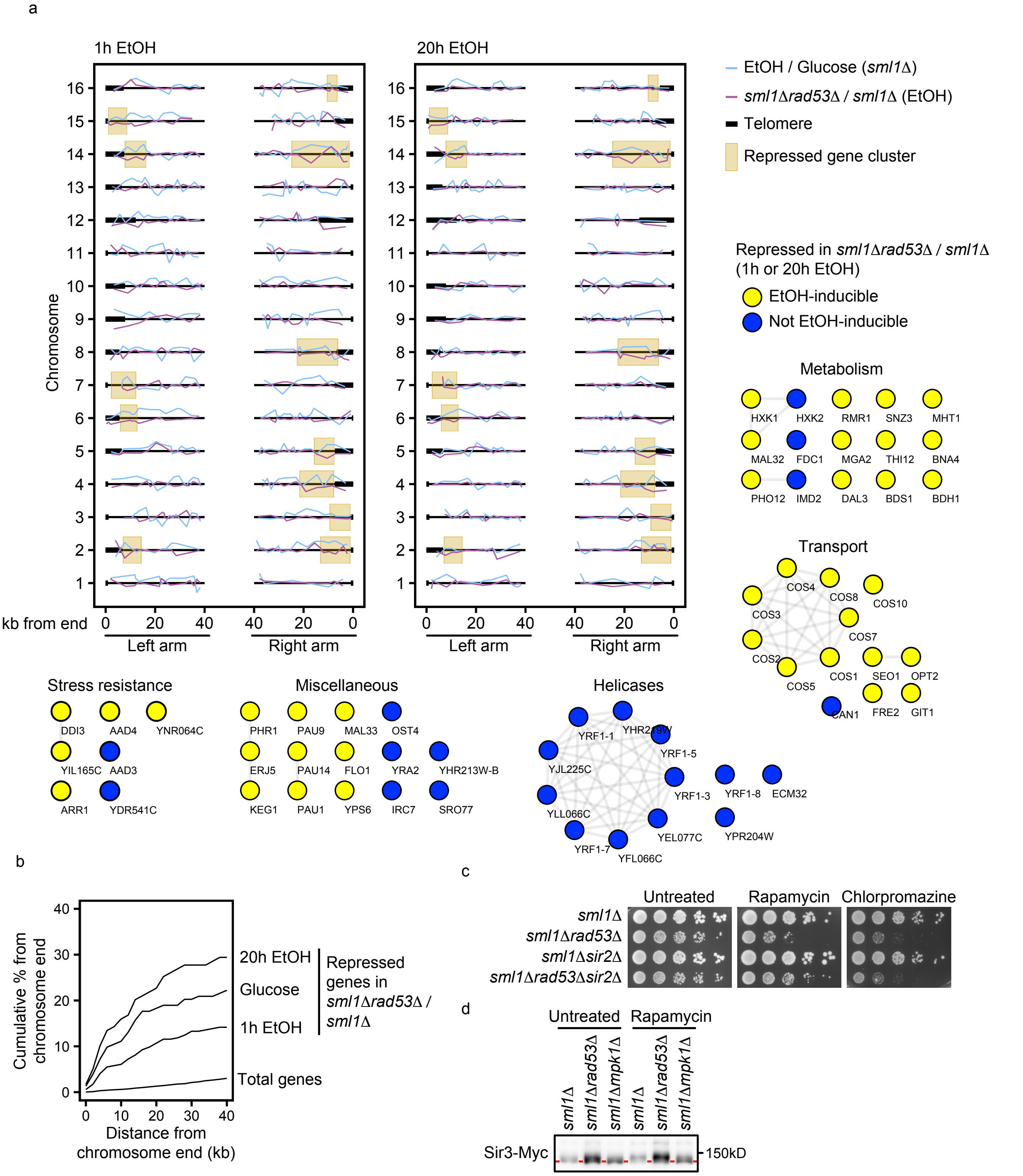

Supplementary Figure 2. Subtelomeric switch response in *rad53Δ* mutants

(a) Depiction of subtelomeric gene expression. The rectangular boxes show the effects of carbon source switch and *RAD53* deletion on gene expression within 40 kb of telomeres. The boxes represent data from 1h (left) or 20h (right) carbon source switch. The chromosomes 1 to 16 are arranged along the vertical axis. For each chromosome, the black baseline represents a 0 fold-change, and the vertical position above or below the baseline represents an up- or down-regulation. Logistic transformation was applied to all fold-changes. Clusters of ethanol-inducible genes that are repressed by *RAD53* deletion (beige-colored boxes) were selected manually for deletion. Bottom/right gene cluster panel: Genes significantly repressed in *sml1Δrad53Δ/sml1Δ* were classified by ethanol inducibility, manually grouped by biological process and clustered by STRING.

(b) Cumulative sum of repressed genes in sml1Δ rad53Δ vs sml1Δ cells cultured in the indicated conditions along the first 40 kb from all telomeres in comparison with the total gene occupancy.

(c) 10^7 cells / mL were serially diluted (1:6), spotted on YP plates with the indicated carbon sources and drugs, and grown for 2d. Rapamycin = 1.5 ng/mL, Chlorpromazine = 10 μM

(d) Cells of the indicated genotypes expressing endogenous 13xMyc-tagged Sir3 were cultured in normal glucose and subjected to 1h treatment with 200 ng/mL rapamycin. Sir3 bandshift indicative of phosphorylation was analyzed by Western blot. The red dashed line indicates un-phosphorylated Sir3.

D = glucose, EtOH = ethanol

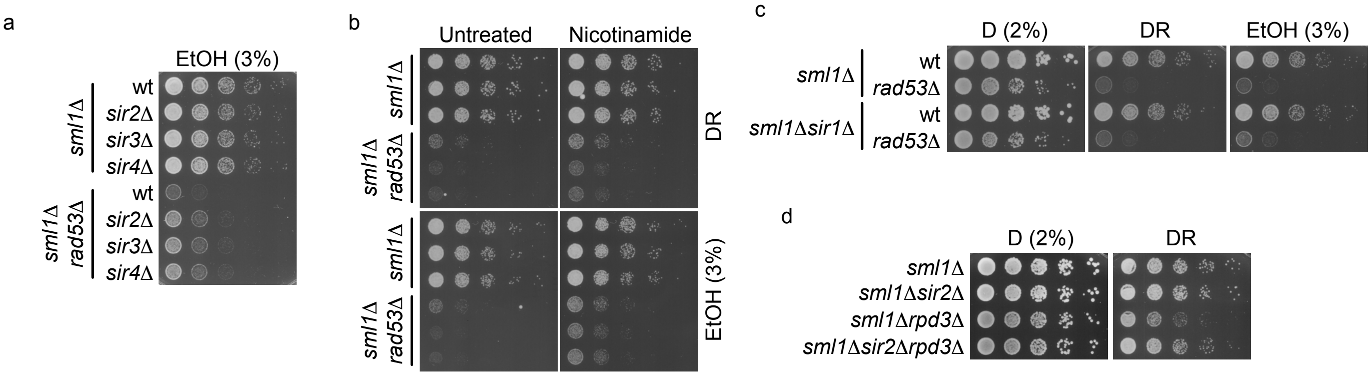

Supplementary Figure 3. Role of the SIR complex in glucose dependence

(a-d) 10^7 cells / mL were serially diluted (1:6), spotted on YP plates with the indicated carbon sources and drugs, and grown for 2d. Nicotinamide = 4 mM

D = glucose, DR = glucose restriction (0.01% in solid media), EtOH = ethanol

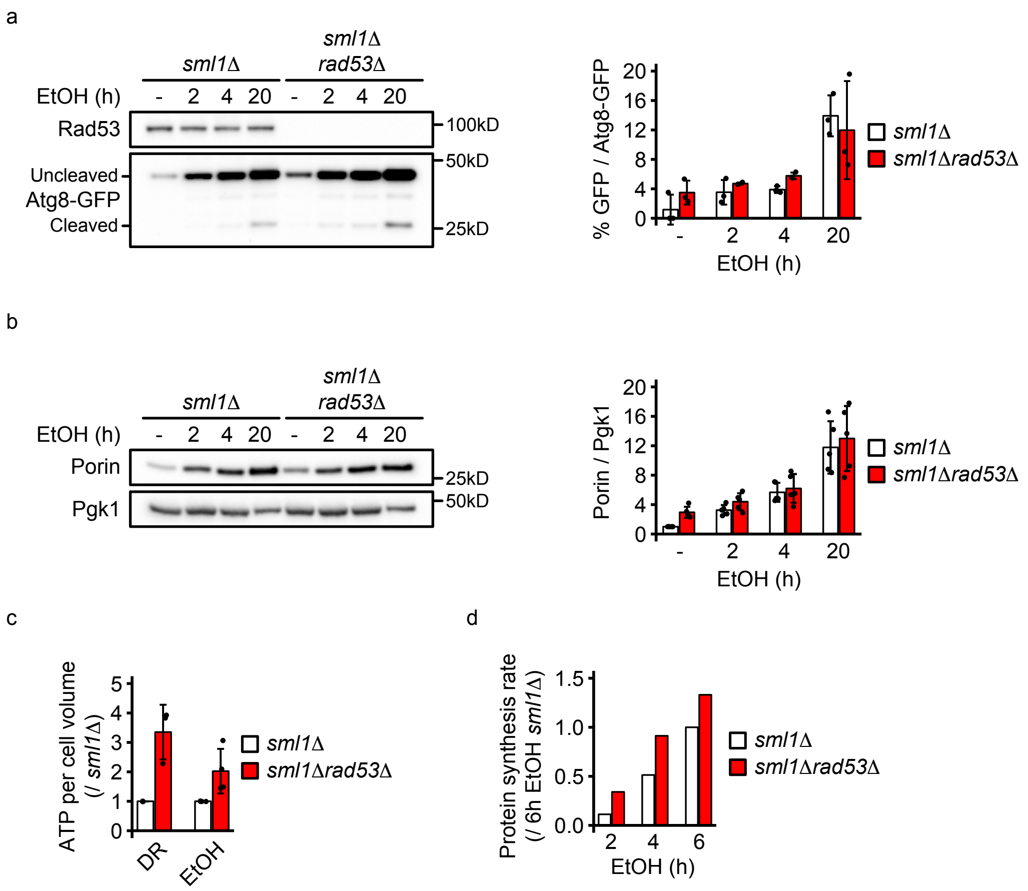

Supplementary Figure 4. Physiological phenotyping of *rad53Δ* mutants
(a) Cells of the indicated genotypes ectopically expressing the Atg8-GFP fusion protein were switched from glucose to ethanol medium for the indicated durations. Protein extracts were prepared and the levels of GFP (cleaved protein) and Atg8-GFP (uncleaved protein) were determined by Western blotting using an anti-GFP antibody. The bar chart in the right panel shows the ratio of cleaved vs. uncleaved protein as measure of autophagy activity. The experiment was performed with 3 independent clones of each genotype.
(b) Cells of the indicated genotypes were switched from glucose to ethanol medium for the indicated durations. Protein extracts were prepared and the level of the mitochondrial protein Porin was determined by Western blotting. The bar chart in the right panel shows the level of Porin normalized to Pgk1. The experiment was performed with 5 independent clones of each genotype.
(c) Cells of the indicated genotypes were adapted to low glucose or ethanol, extracts were prepared from from 1.5 mL culture at ~ $2x10^6$ cells / mL by zirconia bead breakage, and relative ATP levels were measured with a luminescence assay (abcam Cat# ab113849). The experiment was performed 3 times in low glucose and 4 times in ethanol. ATP levels were normalized to cell number and cell volume as measured with a CASY Ton cell counter device. ATP / cell volume was normalized to the *sml1Δ* control of the respective experiment.
(d) Cells of the indicated genotypes were cultured to logarithmic phase in normal glucose and switched to 3% ethanol in Methionine-free synthetic complete medium. After 1h, 3h or 5h, the Methionine analog L-homopropargylglycine was added for 1h at 50 μM. Fixation and staining were performed according to the manufacturer's instructions (ThermoFisher Cat# C10428). Total Methionine analog incorporation was quantified by flow cytometry as measure of protein synthesis rate.

Bars plots with error bars represent mean values and standard deviation, DR = glucose restriction (0.04% in liquid media), EtOH = ethanol

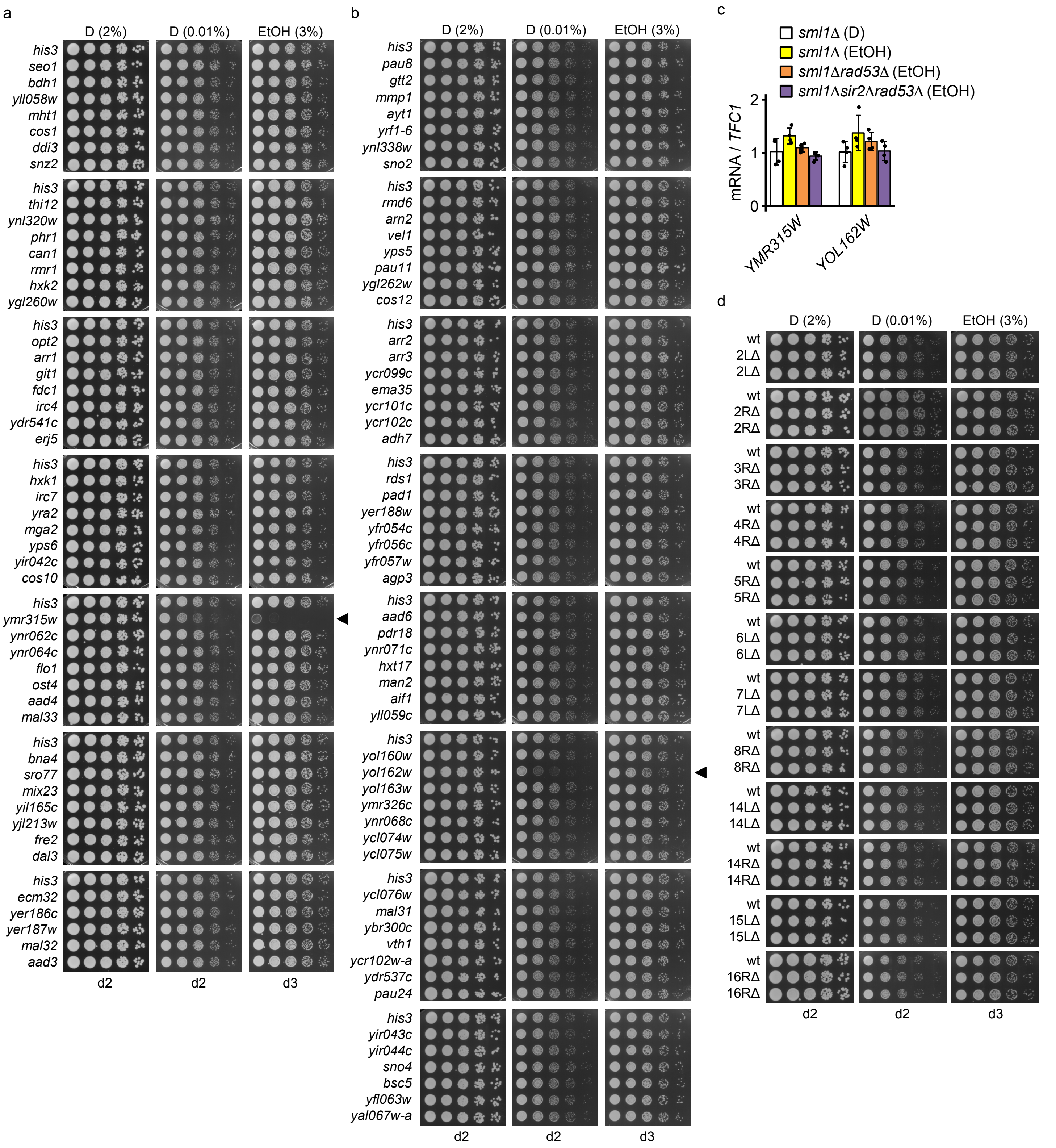

Supplementary Figure 5. Screen for subtelomeric glucose starvation tolerance genes

(a-b) 102 strains covering all mutants with deletions of genes in telomere proximity from the haploid synthetic genetic array library were selected and glucose dependence was determined by spot assay. 10^7 cells / mL were serially diluted (1:6), spotted on YP plates with the indicated carbon sources, and grown for 2 or 3 days as indicated. The arrowheads indicate mutants with slow growth in low glucose or ethanol.

(c) RT-qPCR quantification of the expression of *YMR315W* and *YOL162W* genes in the indicated genotypes and carbon sources. *TFC1* was used as normalization control. n = 4 independent replicate cultures.

(d) *sml1Δ* strains containing deletions of subtelomeric gene clusters depicted in Supplementary Figure 2a. Each deletion strain is named after the chromosome number and arm of the deleted gene cluster. Two independent clones were deletion were analyzed for glucose dependence by spot assay. 10^7 cells / mL were serially diluted (1:6), spotted on YP plates with the indicated carbon sources, and grown for 2 or 3 days as indicated.

Bar plots with error bars represent mean values and standard deviation, D = glucose, DR = glucose restriction (0.01% in solid media), EtOH = ethanol

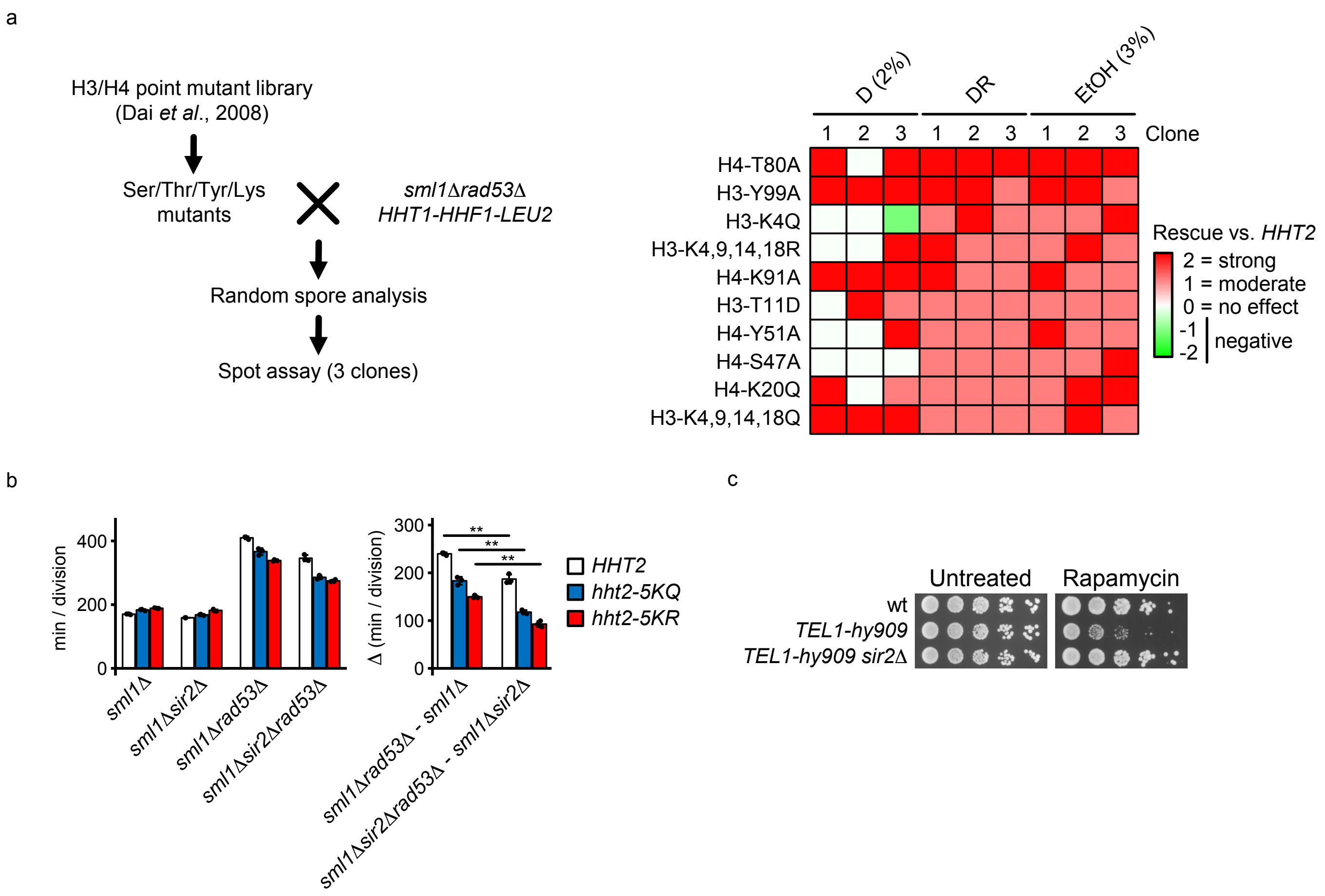

Supplementary Figure 6. Histone acetylation mediates glucose dependence
(a) Screening for functional histone modification sites mediating glucose dependence. Mutants of potentially modifiable amino acids in H3 and H4 were selected from the indicated library and crossed with a *sml1Δrad53Δ HHT1-HHF1-LEU2* query strain. Diploids were sporulated and *sml1Δrad53Δ HHT1-HHF1-LEU2* clones with individual histone point mutations were obtained by random spore analysis. Sensitivity to low glucose and ethanol of 3 independent clones per genotype were analyzed by spot assay, and the genetic interaction relative to the histone wild-type control was assessed visually (right panel). All strains contain the wild-type *HHT1-HHF1* locus with the *LEU2* selection marker integrated in proximity.
(b) Division times of strains in Figure 4a in liquid culture. Cells were inoculated in YP + low glucose and division speed was determined by cell counting over 2 days. Significance was calculated by Student's t-test (two-sided, unpaired, $p_{HHT2} = 0.0070$, $p_{hht2\text{-}5KQ} = 0.0012$, $p_{hht2\text{-}5KR} = 0.0011$). n = 3 independent experiments.
(c) 10^7 cells / mL were serially diluted (1:6), spotted on YPD plates with or without 1.5 ng/mL rapamycin and grown for 2d.

Bars plots with error bars represent mean values and standard deviation, D = glucose, DR = glucose restriction (0.01% in solid media, 0.04% in liquid media), EtOH = ethanol. ** $p < 0.01$

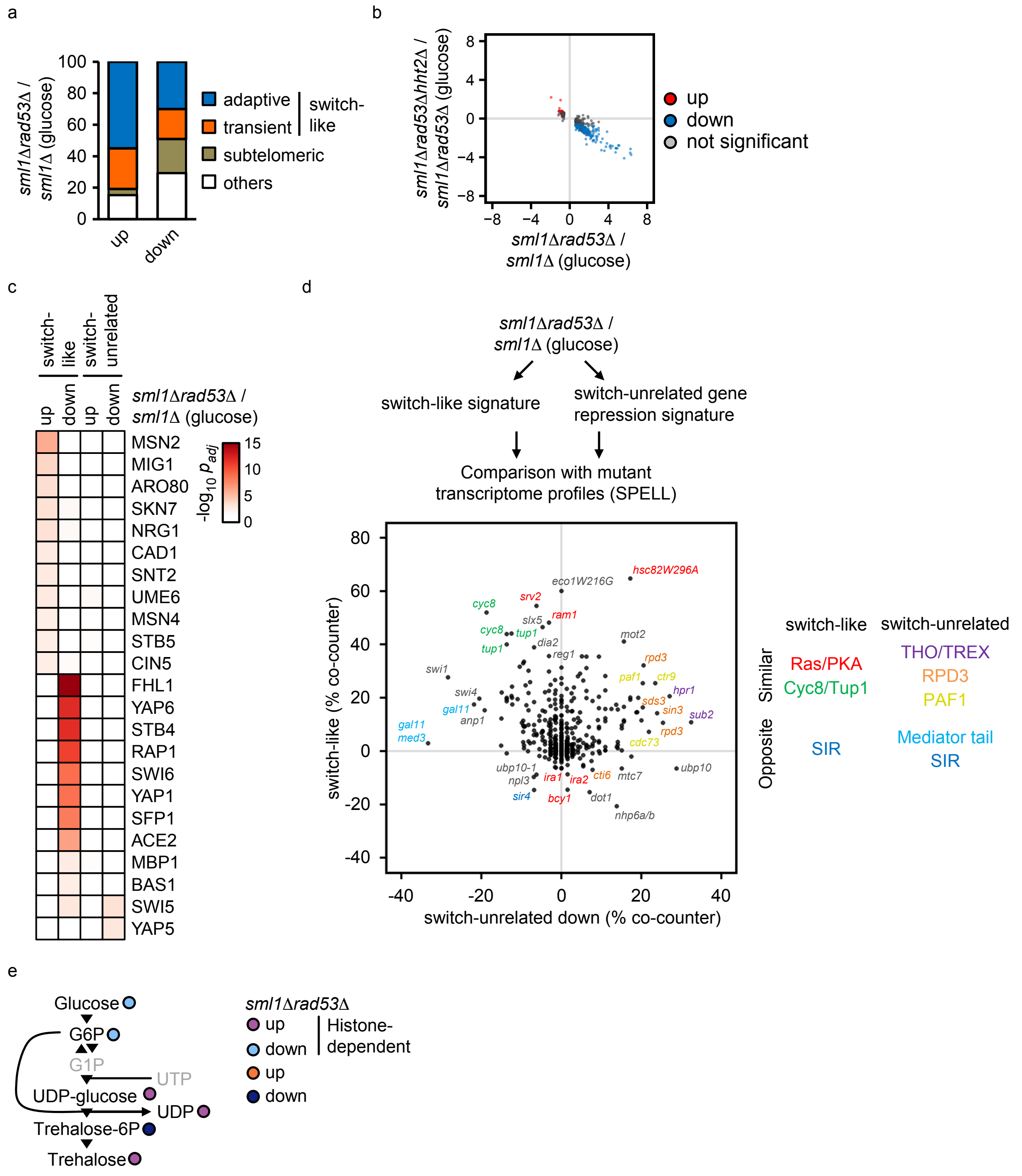

Supplementary Figure 7. Metabolic phenotyping of *rad53Δ* mutants
(a) Classification of differentially expressed genes in *sml1Δrad53Δ* vs. *sml1Δ* cells cultured in 2% glucose by carbon source switch signature and telomere proximity. The RNA-Seq data used for this analysis is presented in Figure 2.
(b) Classification of switch-like differentially expressed genes in *sml1Δrad53Δ* vs. *sml1Δ* cells by *HHT2* dependence.
(c) Transcription factor target enrichment analysis within the indicated gene signatures by hypergeometric test with Benjamini-Hochberg correction. Transcription factor target annotation was obtained from Beyer *et al.*, 2006 [1].
(d) Transcriptome profile intersection analysis with similar literature datasets suggested by the SPELL tool [2].
(e) Classification of altered metabolites related to trehalose synthesis in *sml1Δrad53Δ* vs. *sml1Δ* cells in normal glucose by *HHT2* dependence.

G6P = glucose 6-phosphate, G1P = glucose 1-phosphate

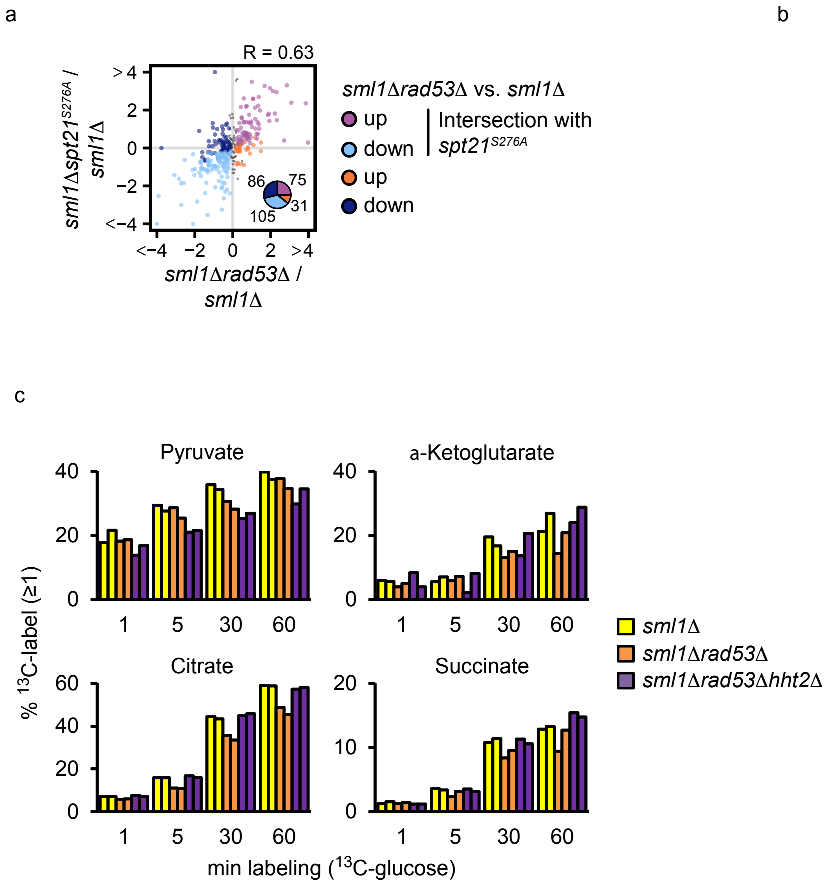

Supplementary Figure 8. Metabolic comparison of Rad53-Spt21 axis mutants and flux analysis
(a) Comparison of altered metabolites in s*ml1Δrad53Δ* vs. *sml1Δ* cells (x axis) and *sml1Δspt21S276A* vs. *sml1Δ* cells (y axis) cultured in normal glucose. The R value shows a high correlation of the fold-changes.
(b) Metabolite alterations in glycolysis and TCA cycle in *sml1Δspt21S276A* vs. *sml1Δ* cells are depicted.
(c) Cells of the indicated genotypes were cultured in synthetic medium with 2% unlabeled glucose in log phase. An equal amount of medium with 2% ^{13}C-glucose (all carbons labeled) was added at 0 min, and samples were quenched at the indicated time points. Metabolites were extracted and labeled vs. total metabolites were quantified by LC/MS analysis.
Ac-CoA = Acetyl-Coenzyme A, G6P = glucose 6-phosphate, G1P = glucose 1-phosphate, F6P = fructose 6-phosphate, F1,6P$_2$ = fructose 1,6-bisphosphate, GA3P = glyceraldehyde 3-phosphate, DHAP = dihydroxyacetone phosphate, G1,3P$_2$ = 1,3-bisphosphoglycerate, 3PG = 3-phosphoglycerate, 2PG = 2-phosphoglycerate, PEP = phosphoenolpyruvate, FA = fatty acids.

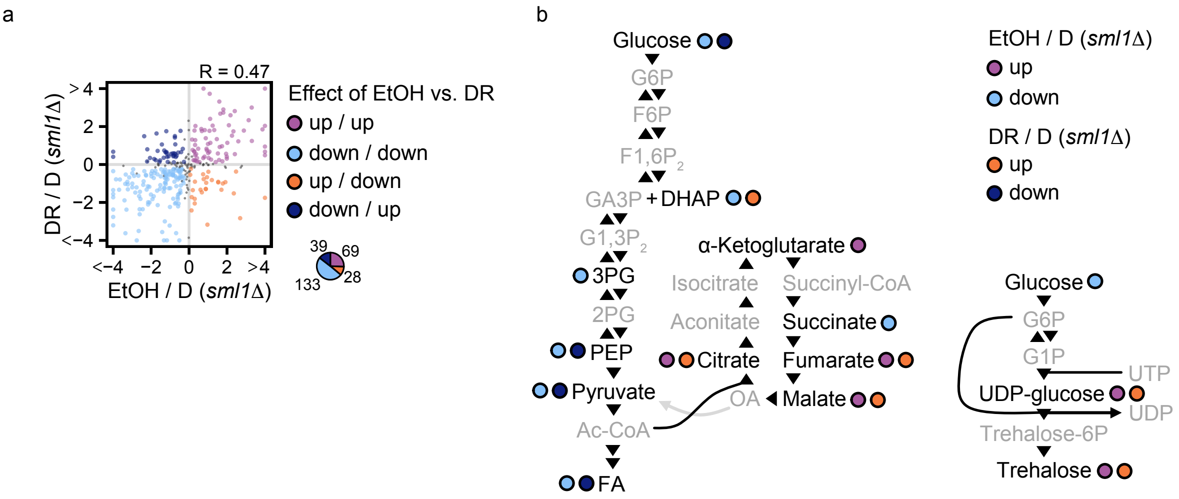

Supplementary Figure 9. Metabolomics of low glucose and ethanol
(a) Comparison of altered metabolites in ethanol vs. normal glucose (x axis) and low glucose vs. normal glucose (x axis) in *sml1Δ* cells. The cells were adapted to the respective carbon source for 20h and harvested during the logarithmic growth phase. The R value shows a high correlation of the fold-changes.
(b) Metabolite alterations in glycolysis, TCA cycle and trehalose metabolism in *sml1Δspt21S276A* vs. *sml1Δ* cells are depicted.

D = glucose, DR = glucose restriction (0.04% in liquid media), EtOH = ethanol, Ac-CoA = Acetyl-Coenzyme A, G6P = glucose 6-phosphate, G1P = glucose 1-phosphate, F6P = fructose 6-phosphate, $F1,6P_2$ = fructose 1,6-bisphosphate, GA3P = glyceraldehyde 3-phosphate, DHAP = dihydroxyacetone phosphate, $G1,3P_2$ = 1,3-bisphosphoglycerate, 3PG = 3-phosphoglycerate, 2PG = 2-phosphoglycerate, PEP = phosphoenolpyruvate, FA = fatty acids.

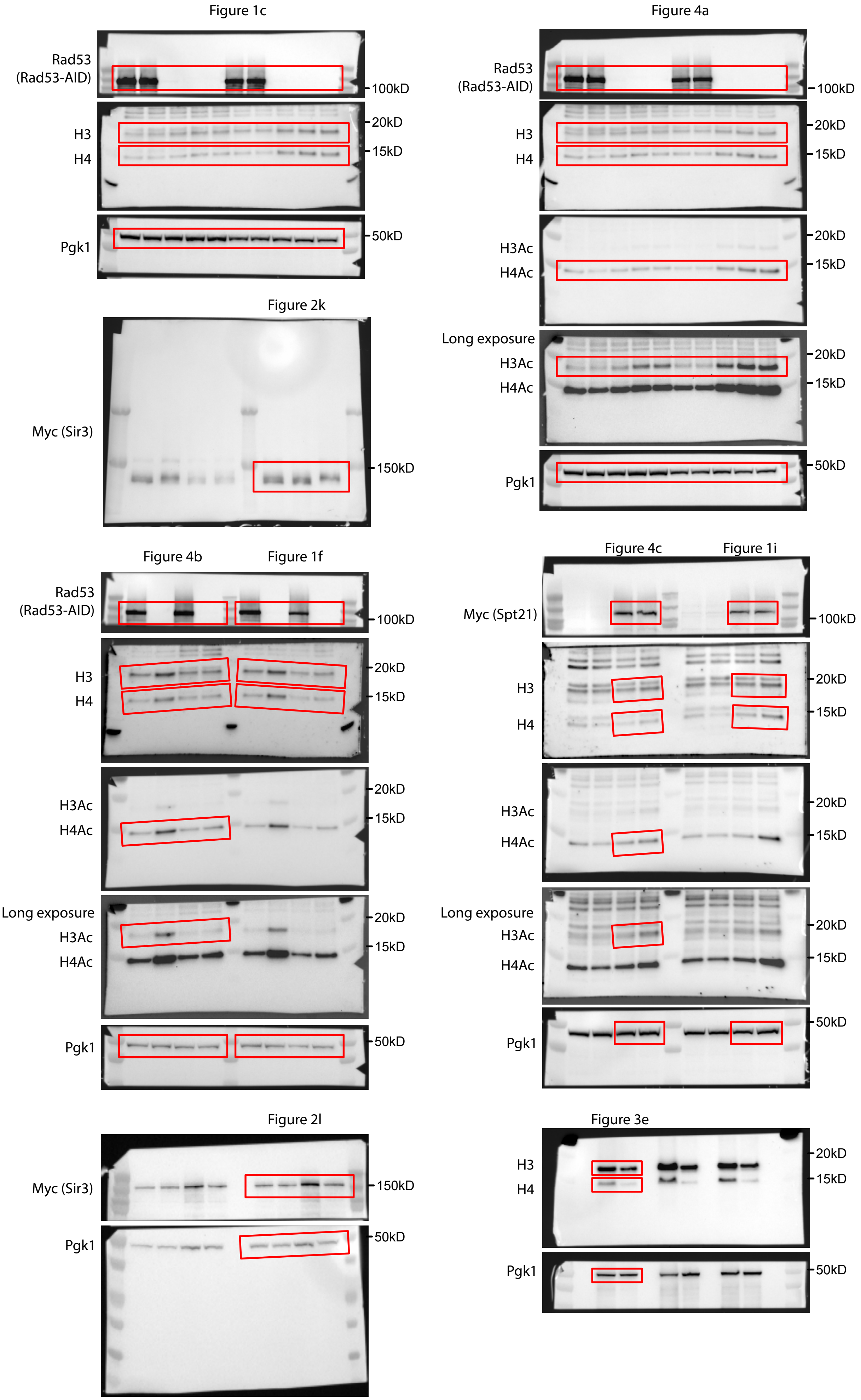

Supplementary Figure 10. Uncropped Western blots from Main Figures

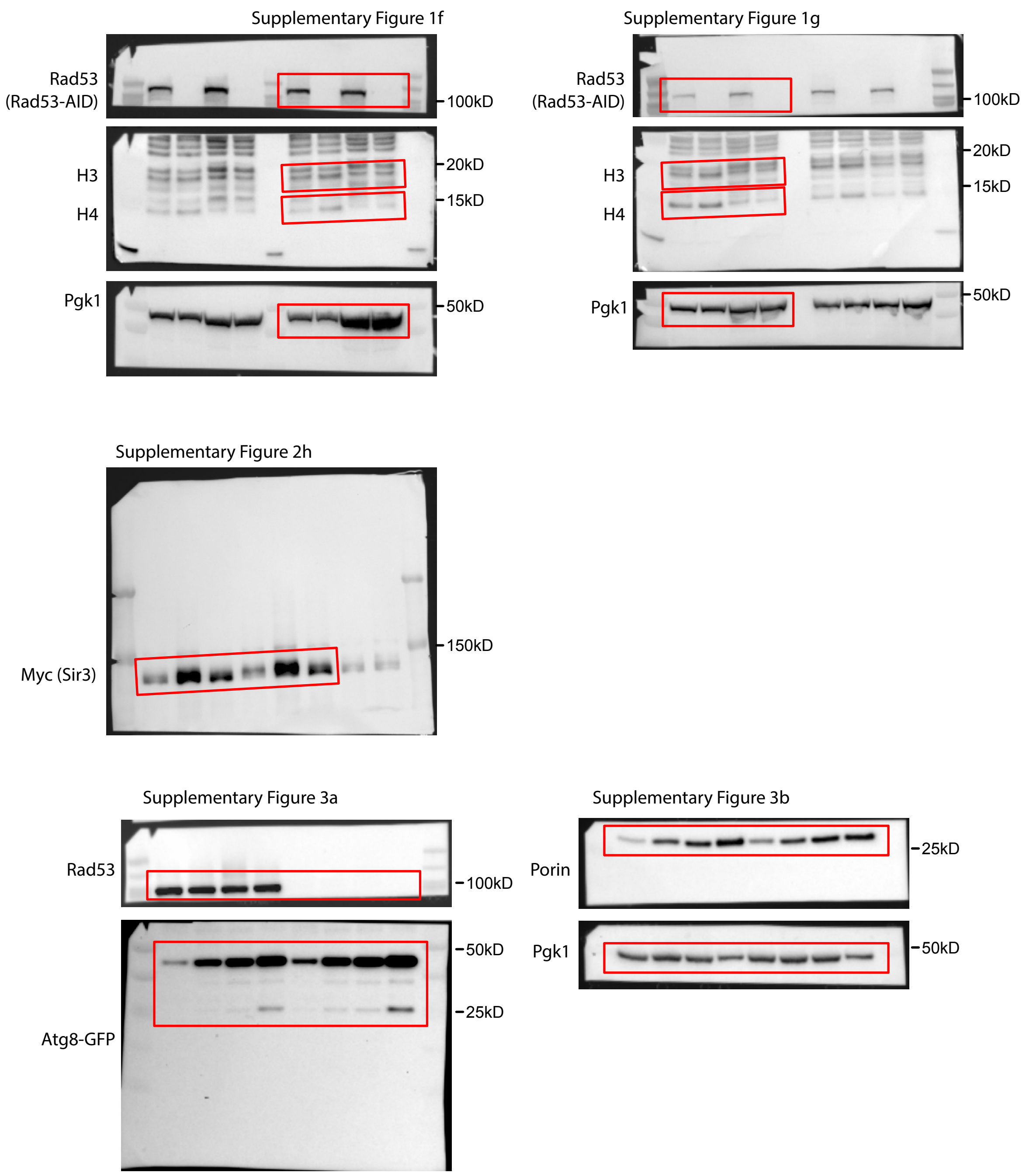

Supplementary Figure 11. Uncropped Western blots from Supplementary Figures

Supplementary Table 1. Rad53-dependent phosphopeptides identified in two SILAC experiments

Gene Name	Rad53-dependent Phosphopeptides	Fold Change (*sml1Δdun1Δhht2Δ* vs. *sml1Δrad53Δhht2Δ*)
CYC8	K.ANSIPSphosVIGAQEPPQEASPAEEATK.A	1.15
ENP1	R.ILDDGSphosNGEDATR.V	-1.01
LAS1	R.M(SS)phosFGTVGKK.N	1.49
MRP8	K.QVSphosELQDLVKK.Q	1.03
NPL3	R.ENSphosLETTFSSVNTR.D	1.09
NUP2	K.QA(SS)phosFSFLNR.A	-1.10
NUP60	R.SNLSphosQENDNEGK.H	1.07
POL1	K.VKIDPDSSTDKYLEIE(SS)phosPLK.L	-1.17
RLP24	K.LVESphosNPELLR.L	1.21
RPL6B	K.QYLSASphosFSLK.N	1.13
SEC16	R.ELSphosEVASR.L	1.27
SPT21	R.TQSphosLPIWNLKPNIANTGFPR.N	979.41

Supplementary Table 2. Yeast strains used in this study

Strain	ID	Genotype	Reference	Clone	Genetic background
wt	SY2080	MAT a, ade2-1, ura3-1, trp1-1, leu2-3, leu2-112, his3-11, his3-15, can1-100, GAL, PSI+, RAD5	Lab collection		W303
tel1Δ	CY14091	MAT a, ade2-1, ura3-1, trp1-1, leu2-3, leu2-112, his3-11, his3-15, can1-100, GAL, PSI+, RAD5, tel1::kanMX6	This study	3	W303
chk1Δ	CY14087	MAT a, ade2-1, ura3-1, trp1-1, leu2-3, leu2-112, his3-11, his3-15, can1-100, GAL, PSI+, RAD5, chk1::natMX6	This study	9	W303
dun1Δ	CY14090	MAT a, ade2-1, ura3-1, trp1-1, leu2-3, leu2-112, his3-11, his3-15, can1-100, GAL, PSI+, RAD5, dun1::natMX6	This study	9	W303
sml1Δ	CY14093	MAT a, ade2-1, ura3-1, trp1-1, leu2-3, leu2-112, his3-11, his3-15, can1-100, GAL, PSI+, RAD5, sml1::hphMX3	This study	2	W303
sml1Δ mec1Δ	CY14095	MAT a, ade2-1, ura3-1, trp1-1, leu2-3, leu2-112, his3-11, his3-15, can1-100, GAL, PSI+, RAD5, sml1::hphMX3, mec1::natMX6	This study	1	W303
sml1Δ rad53Δ	CY14098	MAT a, ade2-1, ura3-1, trp1-1, leu2-3, leu2-112, his3-11, his3-15, can1-100, GAL, PSI+, RAD5, sml1::hphMX3, rad53::natMX6	This study	3	W303
wt for rad53K227A	CY15396	MAT a, ade2-1, ura3-1, trp1-1, leu2-3, leu2-112, his3-11, his3-15, can1-100, GAL, PSI+, RAD5	This study	2	W303
rad53K227A	CY15400	MAT a, ade2-1, ura3-1, trp1-1, leu2-3, leu2-112, his3-11, his3-15, can1-100, GAL, PSI+, RAD5, rad53K227A kan	This study	2	W303
sml1Δ for sml1Δ rad53K227A	CY15398	MAT a, ade2-1, ura3-1, trp1-1, leu2-3, leu2-112, his3-11, his3-15, can1-100, GAL, PSI+, RAD5, sml1::hphMX3	This study	2	W303
sml1Δ rad53K227A	CY15402	MAT a, ade2-1, ura3-1, trp1-1, leu2-3, leu2-112, his3-11, his3-15, can1-100, GAL, PSI+, RAD5, sml1::hphMX3, rad53K227A kan	This study	2	W303

sml1Δ tel1Δ mec1Δ	CB18-79	MAT a, ade2-1, ura3-1, trp1-1, leu2-3, leu2-112, his3-11, his3-15, can1-100, GAL, PSI+, RAD5, sml1::hphMX3, tel1::HIS3, mec1::URA3	This study	2	W303
sml1Δ hht2Δ	CY15171	MAT a, ade2-1, ura3-1, trp1-1, leu2-3, leu2-112, his3-11, his3-15, can1-100, GAL, PSI+, RAD5, sml1::hphMX3, hht2::kanMX6	This study	5	W303
sml1Δ rad53Δ hht2Δ	CB1-78	MAT a, ade2-1, ura3-1, trp1-1, leu2-3, leu2-112, his3-11, his3-15, can1-100, GAL, PSI+, RAD5, sml1::hphMX3, rad53::natMX6, hht2::kanMX6	This study	1	W303
sml1Δ tel1Δ mec1Δ hht2Δ	CB19-02	MAT a, ade2-1, ura3-1, trp1-1, leu2-3, leu2-112, his3-11, his3-15, can1-100, GAL, PSI+, RAD5, sml1::hphMX3, tel1::HIS3, mec1::URA3, hht2::kanMX6	This study	2	W303
sml1Δ RAD53-AID	CY15234	MAT a, ade2-1, ura3-1, trp1-1, leu2-3, leu2-112, his3-11, his3-15, can1-100, GAL, PSI+, RAD5, sml1::URA3, leu2::GPD1-OsTIR::LEU2, rad53::pTDH3-tc3-3xHA-RAD53(kanMX)-AID*-9xMyc::hphMX	This study	1	W303
sml1Δ RAD53-AID spt21Δ	CB8-37	MAT a, ade2-1, ura3-1, trp1-1, leu2-3, leu2-112, his3-11, his3-15, can1-100, GAL, PSI+, RAD5, sml1::URA3, leu2::GPD1-OsTIR::LEU2, rad53::pTDH3-tc3-3xHA-RAD53(kanMX)-AID*-9xMyc::hphMX, spt21::HIS3	This study	1	W303
sml1Δ spt21Δ	CY15162	MAT a, ade2-1, ura3-1, trp1-1, leu2-3, leu2-112, his3-11, his3-15, can1-100, GAL, PSI+, RAD5, sml1::hphMX3, spt21::kanMX6	This study	9	W303
sml1Δ rad53Δ spt21Δ	CY15164	MAT a, ade2-1, ura3-1, trp1-1, leu2-3, leu2-112, his3-11, his3-15, can1-100, GAL, PSI+, RAD5, sml1::hphMX3, rad53::natMX6, spt21::kanMX6	This study	1	W303
sml1Δ spt21-S276A	CB15-35	MAT a, ade2-1, ura3-1, trp1-1, leu2-3, leu2-112, his3-11, his3-15, can1-100, GAL, PSI+, RAD5, sml1::hphMX3, spt21-S276A	This study	1	W303
sml1Δ spt21-S276A hht2Δ	CB17-16	MAT a, ade2-1, ura3-1, trp1-1, leu2-3, leu2-112, his3-11, his3-15, can1-100, GAL, PSI+, RAD5, sml1::hphMX3, spt21-S276A, hht2::kanMX6	This study	1	W303
sml1Δ rad53Δ spt21-S276A	CB15-69	MAT a, ade2-1, ura3-1, trp1-1, leu2-3, leu2-112, his3-11, his3-15, can1-100, GAL, PSI+, RAD5, sml1::hphMX3, spt21-S276A, rad53::natMX6	This study	1	W303

sml1Δ rad53Δ spt21-S276A hht2Δ	CB17-21	MAT alpha, ade2-1, ura3-1, trp1-1, leu2-3, leu2-112, his3-11, his3-15, can1-100, GAL, PSI+, RAD5, sml1::hphMX3, spt21-S276A, rad53::natMX6, hht2::kanMX6	This study	5	W303
sml1Δ SPT21-AID	CB18-21	MAT a, ade2-1, ura3-1, trp1-1, leu2-3, leu2-112, his3-11, his3-15, can1-100, GAL, PSI+, RAD5, sml1::URA3, leu2::GPD1-OsTIR::LEU2, spt21::SPT21-AID*-9xMyc::kanMX	This study	1	W303
sml1Δ spt21-S276A-AID	CB18-24	MAT a, ade2-1, ura3-1, trp1-1, leu2-3, leu2-112, his3-11, his3-15, can1-100, GAL, PSI+, RAD5, sml1::URA3, leu2::GPD1-OsTIR::LEU2, spt21::spt21-S276A-AID*-9xMyc::kanMX	This study	1	W303
sml1Δ rad53Δ hht2Δ for SILAC	CB10-22	MAT a, ade2-1, ura3-1, trp1-1, leu2-3, leu2-112, his3-11, his3-15, CAN1, GAL, PSI+, RAD5, sml1::hphMX3, arg4::natMX6, lys1::URA3, car1::HIS3, rad53::natMX6, hht2::kanMX6	This study	4.5	W303
sml1Δ dun1Δ hht2Δ for SILAC	CB10-27	MAT a, ade2-1, ura3-1, trp1-1, leu2-3, leu2-112, his3-11, his3-15, CAN1, GAL, PSI+, RAD5, sml1::hphMX3, arg4::natMX6, lys1::URA3, car1::HIS3, dun1::natMX6, hht2::kanMX6	This study	4.1	W303
sml1Δ RAD53-AID spt10Δ	CB15-17	MAT a, ade2-1, ura3-1, trp1-1, leu2-3, leu2-112, his3-11, his3-15, can1-100, GAL, PSI+, RAD5, sml1::URA3, leu2::GPD1-OsTIR::LEU2, rad53::pTDH3-tc3-3xHA-RAD53(kanMX)-AID*-9xMyc::hphMX, spt10::HIS3	This study	1	W303
sml1Δ TEL-5R	CB10-36	Mat alpha, TRP1, ura3Δ0, leu2Δ0, his3Δ1, lys2Δ202, can1::HPHMX, ade2-661, yer188w::112tetO-URA3, lys2Δ202::tetR-GFP-LYS2, pNUP49-GFP-NOP1 mCherry-ADE2, sml1::kanMX6	This study	2	S288C
sml1Δ rad53Δ TEL-5R	CB15-45	Mat alpha, TRP1, ura3Δ0, leu2Δ0, his3Δ1, lys2Δ202, can1::HPHMX, ade2-661, yer188w::112tetO-URA3, lys2Δ202::tetR-GFP-LYS2, pNUP49-GFP-NOP1 mCherry-ADE2, sml1::kanMX6, rad53::natMX6	This study	2	S288C
sml1Δ rad53Δ TEL-5R	CB15-47	Mat alpha, TRP1, ura3Δ0, leu2Δ0, his3Δ1, lys2Δ202, can1::HPHMX, ade2-661, yer188w::112tetO-URA3, lys2Δ202::tetR-GFP-LYS2, pNUP49-GFP-NOP1 mCherry-ADE2, sml1::kanMX6, rad53::natMX6	This study	4	S288C
sml1Δ TEL-14R	CB10-35	Mat alpha, TRP1, ura3Δ0, leu2Δ0, his3Δ1, lys2Δ202, can1::HPHMX, ade2-661,	This study	2	S288C

		ynr075w::112tetO-URA3, lys2Δ202::tetR-GFP-LYS2, pNUP49-GFP-NOP1 mCherry-ADE2, sml1::kanMX6			
sml1Δ rad53Δ TEL-14R	CB15-40	Mat alpha, TRP1, ura3Δ0, leu2Δ0, his3Δ1, lys2Δ202, can1::HPHMX, ade2-661, ynr075w::112tetO-URA3, lys2Δ202::tetR-GFP-LYS2, pNUP49-GFP-NOP1 mCherry-ADE2, sml1::kanMX6, rad53::natMX6	This study	1	S288C
sml1Δ rad53Δ TEL-14R	CB15-41	Mat alpha, TRP1, ura3Δ0, leu2Δ0, his3Δ1, lys2Δ202, can1::HPHMX, ade2-661, ynr075w::112tetO-URA3, lys2Δ202::tetR-GFP-LYS2, pNUP49-GFP-NOP1 mCherry-ADE2, sml1::kanMX6, rad53::natMX6	This study	2	S288C
sml1Δ sir2Δ	CB2-81	MAT a, ade2-1, ura3-1, trp1-1, leu2-3, leu2-112, his3-11, his3-15, can1-100, GAL, PSI+, RAD5, sml1::hphMX3, sir2::kanMX6	This study	1	W303
sml1Δ sir3Δ	CB3-6	MAT a, ade2-1, ura3-1, trp1-1, leu2-3, leu2-112, his3-11, his3-15, can1-100, GAL, PSI+, RAD5, sml1::hphMX3, sir3::kanMX6	This study	1	W303
sml1Δ sir4Δ	CB3-12	MAT a, ade2-1, ura3-1, trp1-1, leu2-3, leu2-112, his3-11, his3-15, can1-100, GAL, PSI+, RAD5, sml1::hphMX3, sir4::kanMX6	This study	1	W303
sml1Δ rad53Δ sir2Δ	CB3-3	MAT a, ade2-1, ura3-1, trp1-1, leu2-3, leu2-112, his3-11, his3-15, can1-100, GAL, PSI+, RAD5, sml1::hphMX3, rad53::natMX6, sir2::kanMX6	This study	4	W303
sml1Δ rad53Δ sir3Δ	CB3-9	MAT a, ade2-1, ura3-1, trp1-1, leu2-3, leu2-112, his3-11, his3-15, can1-100, GAL, PSI+, RAD5, sml1::hphMX3, rad53::natMX6, sir3::kanMX6	This study	1	W303
sml1Δ rad53Δ sir4Δ	CB3-15	MAT a, ade2-1, ura3-1, trp1-1, leu2-3, leu2-112, his3-11, his3-15, can1-100, GAL, PSI+, RAD5, sml1::hphMX3, rad53::natMX6, sir4::kanMX6	This study	2	W303
sml1Δ sir1Δ	CB2-75	MAT a, ade2-1, ura3-1, trp1-1, leu2-3, leu2-112, his3-11, his3-15, can1-100, GAL, PSI+, RAD5, sml1::hphMX3, sir1::kanMX6	This study	2	W303
sml1Δ rad53Δ sir1Δ	CB2-79	MAT a, ade2-1, ura3-1, trp1-1, leu2-3, leu2-112, his3-11, his3-15, can1-100, GAL, PSI+, RAD5, sml1::hphMX3, rad53::natMX6, sir1::kanMX6	This study	6	W303
sml1Δ rpd3Δ	CB16-26	MAT a, ade2-1, ura3-1, trp1-1, leu2-3, leu2-112, his3-11, his3-15, can1-100, GAL, PSI+, RAD5, sml1::hphMX3, rpd3::HIS3	This study	3	W303

sml1Δ rpd3Δ sir2Δ	CB16-30	MAT a, ade2-1, ura3-1, trp1-1, leu2-3, leu2-112, his3-11, his3-15, can1-100, GAL, PSI+, RAD5, sml1::hphMX3, sir2::kanMX6, rpd3::HIS3	This study	3	W303
sml1Δ hht2-5KQ	CB16-65	MAT a, ade2-1, ura3-1, trp1-1, leu2-3, leu2-112, his3-11, his3-15, can1-100, GAL, PSI+, RAD5, sml1::hphMX3, hht2-K4,9,14,18,27Q	This study	4	W303
sml1Δ hht2-5KQ rad53Δ	CB17-08	MAT a, ade2-1, ura3-1, trp1-1, leu2-3, leu2-112, his3-11, his3-15, can1-100, GAL, PSI+, RAD5, sml1::hphMX3, hht2-K4,9,14,18,27Q, rad53::natMX6	This study	2	W303
sml1Δ hht2-5KQ sir2Δ	CB17-35	MAT a, ade2-1, ura3-1, trp1-1, leu2-3, leu2-112, his3-11, his3-15, can1-100, GAL, PSI+, RAD5, sml1::hphMX3, hht2-K4,9,14,18,27Q, sir2::kanMX6	This study	1	W303
sml1Δ hht2-5KQ sir2Δ rad53Δ	CB18-09	MAT a, ade2-1, ura3-1, trp1-1, leu2-3, leu2-112, his3-11, his3-15, can1-100, GAL, PSI+, RAD5, sml1::hphMX3, hht2-K4,9,14,18,27Q, rad53::natMX6, sir2::kanMX6	This study	1	W303
sml1Δ hht2-5KR	CB16-69	MAT a, ade2-1, ura3-1, trp1-1, leu2-3, leu2-112, his3-11, his3-15, can1-100, GAL, PSI+, RAD5, sml1::hphMX3, hht2-K4,9,14,18,27R	This study	1	W303
sml1Δ hht2-5KR rad53Δ	CB17-11	MAT a, ade2-1, ura3-1, trp1-1, leu2-3, leu2-112, his3-11, his3-15, can1-100, GAL, PSI+, RAD5, sml1::hphMX3, hht2-K4,9,14,18,27R, rad53::natMX6	This study	2	W303
sml1Δ hht2-5KR sir2Δ	CB17-39	MAT a, ade2-1, ura3-1, trp1-1, leu2-3, leu2-112, his3-11, his3-15, can1-100, GAL, PSI+, RAD5, sml1::hphMX3, hht2-K4,9,14,18,27R, sir2::kanMX6	This study	1	W303
sml1Δ hht2-5KR sir2Δ rad53Δ	CB18-13	MAT a, ade2-1, ura3-1, trp1-1, leu2-3, leu2-112, his3-11, his3-15, can1-100, GAL, PSI+, RAD5, sml1::hphMX3, hht2-K4,9,14,18,27R, rad53::natMX6, sir2::kanMX6	This study	1	W303
sml1Δ rad53Δ rpd3Δ	CB16-34	MAT a, ade2-1, ura3-1, trp1-1, leu2-3, leu2-112, his3-11, his3-15, can1-100, GAL, PSI+, RAD5, sml1::hphMX3, rad53::natMX6, rpd3::HIS3	This study	3	W303
sml1Δ rad53Δ rpd3Δ sir2Δ	CB16-38	MAT a, ade2-1, ura3-1, trp1-1, leu2-3, leu2-112, his3-11, his3-15, can1-100, GAL, PSI+, RAD5, sml1::hphMX3, rad53::natMX6, sir2::kanMX6, rpd3::HIS3	This study	3	W303

TEL1-hy909	Sy2633	MAT a, ade2-1, ura3-1, trp1-1, leu2-3, leu2-112, his3-11, his3-15, can1-100, GAL, PSI+, RAD5, TEL1-hy909::LEU2	Baldo et al., 2008		W303
TEL1-hy909 sir2Δ	CB5-75	MAT a, ade2-1, ura3-1, trp1-1, leu2-3, leu2-112, his3-11, his3-15, can1-100, GAL, PSI+, RAD5, TEL1-hy909::LEU2, sir2::kanMX6	This study	1	W303
TEL1-hy909 sir2Δ	CB5-76	MAT a, ade2-1, ura3-1, trp1-1, leu2-3, leu2-112, his3-11, his3-15, can1-100, GAL, PSI+, RAD5, TEL1-hy909::LEU2, sir2::kanMX6	This study	2	W303
TEL1-hy909 hht2Δ	CB5-78	MAT a, ade2-1, ura3-1, trp1-1, leu2-3, leu2-112, his3-11, his3-15, can1-100, GAL, PSI+, RAD5, TEL1-hy909::LEU2, hht2::kanMX6	This study	1	W303
TEL1-hy909 hht2Δ	CB5-79	MAT a, ade2-1, ura3-1, trp1-1, leu2-3, leu2-112, his3-11, his3-15, can1-100, GAL, PSI+, RAD5, TEL1-hy909::LEU2, hht2::kanMX6	This study	2	W303
rpd3Δ	CB9-41	MAT a, ade2-1, ura3-1, trp1-1, leu2-3, leu2-112, his3-11, his3-15, can1-100, GAL, PSI+, RAD5, rpd3::HIS3	This study	1	W303
TEL1-hy909 rpd3Δ	CB9-45	MAT a, ade2-1, ura3-1, trp1-1, leu2-3, leu2-112, his3-11, his3-15, can1-100, GAL, PSI+, RAD5, TEL1-hy909::LEU2, rpd3::HIS3	This study	2	W303
sml1Δ rad53Δ HHT1-HHF1-LEU2 (query)	CB7-67	MAT alpha, can1::STE2pr-Sp_his5, lyp1Δ, ura3Δ0, his3Δ1, met15Δ0, leu2Δ0, sml1::URA3, rad53::kanMX6, HHT1-HHF1-LEU2	This study	1	S288C
SIR3-13Myc sml1Δ	CB-SIR3Myc_S1	MAT a, ade2-1, ura3-1, trp1-1, leu2-3, leu2-112, his3-11, his3-15, can1-100, GAL, PSI+, RAD5, SIR3::SIR3-13Myc-kanMX6, sml1::hphMX3	This study	1	W303
SIR3-13Myc sml1Δ hht2Δ	CB-SIR3Myc_SH1	MAT a, ade2-1, ura3-1, trp1-1, leu2-3, leu2-112, his3-11, his3-15, can1-100, GAL, PSI+, RAD5, SIR3::SIR3-13Myc-kanMX6, sml1::hphMX3, hht2::kanMX6	This study	1	W303
SIR3-13Myc sml1Δ rad53Δ	CB-SIR3Myc_SR1	MAT a, ade2-1, ura3-1, trp1-1, leu2-3, leu2-112, his3-11, his3-15, can1-100, GAL, PSI+, RAD5, SIR3::SIR3-13Myc-kanMX6, sml1::hphMX3, rad53::natMX6	This study	1	W303
SIR3-13Myc sml1Δ	CB-SIR3Myc_SRH1	MAT a, ade2-1, ura3-1, trp1-1, leu2-3, leu2-112, his3-11, his3-15, can1-100, GAL, PSI+, RAD5,	This study	1	W303

rad53Δ hht2Δ		SIR3::SIR3-13Myc-kanMX6, sml1::hphMX3, hht2::kanMX6, rad53::natMX6			
SIR3-13Myc sml1Δ mpk1Δ	CB-SIR3Myc_SM1	MAT a, ade2-1, ura3-1, trp1-1, leu2-3, leu2-112, his3-11, his3-15, can1-100, GAL, PSI+, RAD5, SIR3::SIR3-13Myc-kanMX6, sml1::hphMX3, mpk1::HIS3	This study	1	W303
sml1Δ ATG8-GFP	CB4-20	MAT a, ade2-1, ura3-1, trp1-1, leu2-3, leu2-112, his3-11, his3-15, can1-100, GAL, PSI+, RAD5, sml1::hphMX3, pRS416 GFP-ATG8-URA3	This study	2	W303
sml1Δ ATG8-GFP	CB4-21	MAT a, ade2-1, ura3-1, trp1-1, leu2-3, leu2-112, his3-11, his3-15, can1-100, GAL, PSI+, RAD5, sml1::hphMX3, pRS416 GFP-ATG8-URA3	This study	4	W303
sml1Δ ATG8-GFP	CB4-22	MAT a, ade2-1, ura3-1, trp1-1, leu2-3, leu2-112, his3-11, his3-15, can1-100, GAL, PSI+, RAD5, sml1::hphMX3, pRS416 GFP-ATG8-URA3	This study	5	W303
sml1Δ rad53Δ ATG8-GFP	CB4-23	MAT a, ade2-1, ura3-1, trp1-1, leu2-3, leu2-112, his3-11, his3-15, can1-100, GAL, PSI+, RAD5, sml1::hphMX3, rad53::natMX6, pRS416 GFP-ATG8-URA3	This study	1	W303
sml1Δ rad53Δ ATG8-GFP	CB4-24	MAT a, ade2-1, ura3-1, trp1-1, leu2-3, leu2-112, his3-11, his3-15, can1-100, GAL, PSI+, RAD5, sml1::hphMX3, rad53::natMX6, pRS416 GFP-ATG8-URA3	This study	3	W303
sml1Δ rad53Δ ATG8-GFP	CB4-25	MAT a, ade2-1, ura3-1, trp1-1, leu2-3, leu2-112, his3-11, his3-15, can1-100, GAL, PSI+, RAD5, sml1::hphMX3, rad53::natMX6, pRS416 GFP-ATG8-URA3	This study	6	W303
2LΔ	CB_2LΔ-#5	MAT a his3Δ1 leu2Δ0 ura3Δ0 met15Δ0 CHROMOSOME2(7605-13879)::kanMX6	This study	5	S288C
2LΔ	CB_2LΔ-#7	MAT a his3Δ1 leu2Δ0 ura3Δ0 met15Δ0 CHROMOSOME2(7605-13879)::kanMX6	This study	7	S288C
2RΔ	CB_2RΔ-#16	MAT a his3Δ1 leu2Δ0 ura3Δ0 met15Δ0 CHROMOSOME2(800523-811479)::kanMX6	This study	16	S288C
2RΔ	CB_2RΔ-#17	MAT a his3Δ1 leu2Δ0 ura3Δ0 met15Δ0 CHROMOSOME2(800523-811479)::kanMX6	This study	17	S288C
3RΔ	CB_3RΔ-#3	MAT a his3Δ1 leu2Δ0 ura3Δ0 met15Δ0 CHROMOSOME3(307801-314981)::kanMX6	This study	3	S288C
3RΔ	CB_3RΔ-#4	MAT a his3Δ1 leu2Δ0 ura3Δ0 met15Δ0 CHROMOSOME3(307801-314981)::kanMX6	This study	4	S288C

4RΔ	CB_4RΔ-#3	MAT a his3Δ1 leu2Δ0 ura3Δ0 met15Δ0 CHROMOSOME4(1510902-1523611)::kanMX6	This study	3	S288C
4RΔ	CB_4RΔ-#4	MAT a his3Δ1 leu2Δ0 ura3Δ0 met15Δ0 CHROMOSOME4(1510902-1523611)::kanMX6	This study	4	S288C
5RΔ	CB_5RΔ-#1	MAT a his3Δ1 leu2Δ0 ura3Δ0 met15Δ0 CHROMOSOME5(561705-568759)::kanMX6	This study	1	S288C
5RΔ	CB_5RΔ-#6	MAT a his3Δ1 leu2Δ0 ura3Δ0 met15Δ0 CHROMOSOME5(561705-568759)::kanMX6	This study	6	S288C
6LΔ	CB_6LΔ-#2	MAT a his3Δ1 leu2Δ0 ura3Δ0 met15Δ0 CHROMOSOME6(6426-12259)::kanMX6	This study	2	S288C
6LΔ	CB_6LΔ-#3	MAT a his3Δ1 leu2Δ0 ura3Δ0 met15Δ0 CHROMOSOME6(6426-12259)::kanMX6	This study	3	S288C
7LΔ	CB_7LΔ-#6	MAT a his3Δ1 leu2Δ0 ura3Δ0 met15Δ0 CHROMOSOME7(2790-11730)::kanMX6	This study	6	S288C
7LΔ	CB_7LΔ-#7	MAT a his3Δ1 leu2Δ0 ura3Δ0 met15Δ0 CHROMOSOME7(2790-11730)::kanMX6	This study	7	S288C
8RΔ	CB_8RΔ-#2	MAT a his3Δ1 leu2Δ0 ura3Δ0 met15Δ0 CHROMOSOME8(540549-555967)::kanMX6	This study	2	S288C
8RΔ	CB_8RΔ-#3	MAT a his3Δ1 leu2Δ0 ura3Δ0 met15Δ0 CHROMOSOME8(540549-555967)::kanMX6	This study	3	S288C
14LΔ	CB_14LΔ-#1	MAT a his3Δ1 leu2Δ0 ura3Δ0 met15Δ0 CHROMOSOME14(760067-782280)::kanMX6	This study	1	S288C
14LΔ	CB_14LΔ-#5	MAT a his3Δ1 leu2Δ0 ura3Δ0 met15Δ0 CHROMOSOME14(760067-782280)::kanMX6	This study	5	S288C
14RΔ	CB_14RΔ-#1	MAT a his3Δ1 leu2Δ0 ura3Δ0 met15Δ0 CHROMOSOME14(8330-15854)::kanMX6	This study	1	S288C
14RΔ	CB_14RΔ-#4	MAT a his3Δ1 leu2Δ0 ura3Δ0 met15Δ0 CHROMOSOME14(8330-15854)::kanMX6	This study	4	S288C
15LΔ	CB_15LΔ-#1	MAT a his3Δ1 leu2Δ0 ura3Δ0 met15Δ0 CHROMOSOME15(1647-8115)::kanMX6	This study	1	S288C
15LΔ	CB_15LΔ-#2	MAT a his3Δ1 leu2Δ0 ura3Δ0 met15Δ0 CHROMOSOME15(1647-8115)::kanMX6	This study	2	S288C
16RΔ	CB_16RΔ-#1	MAT a his3Δ1 leu2Δ0 ura3Δ0 met15Δ0 CHROMOSOME16(938148-941136)::kanMX6	This study	1	S288C
16RΔ	CB_16RΔ-#2	MAT a his3Δ1 leu2Δ0 ura3Δ0 met15Δ0 CHROMOSOME16(938148-941136)::kanMX6	This study	2	S288C

Supplementary Table 3. Primers used in this study

Primer Name	Primer Sequence
TFC1-F qPCR	CCAAGCTCATGAACAAGTCC
TFC1-R qPCR	ACCCAAATTGGACGTCTAGC
HHT2-F qPCR	CTTCTGCTATCGGTGCTTTG
HHT2-R qPCR	CGTGAATAGCAGCCAGATTAGTG
HHF2-F qPCR	TCTAGGAAAAGGTGGTGCCAAG
HHF2-R qPCR	CTTCTGATAGCTGGCTTAGTG
COS1-F qPCR	CACGGGGAATGTGCCTTC
COS1-R qPCR	GCATCTTGTGTTCCATTTTCACG
COS8-F qPCR	AAAGACCGTTCTACCTCAAGATG
COS8-R qPCR	AGTGGTAGCCATAACAGCATCC
YMR315W-F qPCR	TGAAAGTTTCGGCGTCAACG
YMR315W-R qPCR	AAAAACGCGTCCACACAAGC
YOL162W-F qPCR	ACGTTTTGGCCACATACCTG
YOL162W-R qPCR	TAGCAGGTTGGCTTGAAAGG

Supplementary Table 4. *q* values from SAM analysis for Figure 6d

name	sml1rad53_D_vs_sml1_D_fc_log2	sml1rad53hht2_D_vs_sml1rad53_D_fc_log2	sml1spt21S276A_D_vs_sml1_D_fc_log2	sml1rad53_D_vs_sml1_D_q	sml1rad53hht2_D_vs_sml1rad53_D_q	sml1spt21S276A_D_vs_sml1_D_q
C6:0	-0.00288	-0.22826	-1.13233	NA	0.21096	0
C8:0	-0.51093	0.354822	-0.95722	0	0.00881	0
C10:0	-1.28358	0.625022	-0.79687	0	0	0
C12:0	-0.75388	0.200772	-0.63206	0	0.0584	0.0217
C14:0	-1.46948	0.826094	-0.66678	0	0.00346	0.2181
C14:1n5	-1.04618	0.369983	-1.06342	0	0.00346	0.00855
C16:0	-1.07115	0.616649	0.091936	0	0.01822	0.3198
C16:1n7	-1.4736	1.105819	-0.61461	0	0	0.2181
C18:0	-0.49555	0.318136	0.115571	0	0.0584	0.3198
C18:1	-1.24803	0.739962	0.088645	0	0.01822	0.3198
C18:2n6	-0.62273	0.399514		0	0.0584	NA
C18:3n3 / C18:3n6	0.13037	-0.38504		0.15711	0.03513	NA
C20:0	-0.63464	0.671807	-0.17589	0.00644	0.01822	0.29292
C20:1	-0.64566	0.745566	0.422063	0.00241	0.01822	0.27199
C22:0	-0.65678	1.169498		0.06367	0.01822	NA
C22:1n9	-0.46221	0.653661	-0.10292	0.01753	0.03513	0.30895

Supplementary Table 5. *q* values from SAM analysis for Figure 6e

name	sml1_EtOH_vs_sml1_D_fc_log2	sml1_EtOH_vs_sml1_D_q	sml1rad53_EtOH_vs_sml1_EtOH_fc_log2	sml1rad53_EtOH_vs_sml1_EtOH_q	sml1_DR_vs_sml1_D_fc_log2	sml1_DR_vs_sml1_D_q	sml1rad53_DR_vs_sml1_DR_fc_log2	sml1rad53_DR_vs_sml1_DR_q	sml1rad53_DR_acetate_vs_sml1rad53_DR_fc_log2	sml1rad53_DR_acetate_vs_sml1rad53_DR_q
C6:0	-0.67122	0.00258	-0.74453	0.02158	-1.14261	0	-0.01481	NA	0.619499	0.00132
C8:0	-1.92815	0	-0.76532	0	-1.82357	0	-0.45387	0	-1.12941	0
C10:0	-3.8494	0	-0.46101	0.00343	-0.78205	0	-1.23675	0	-0.45224	0
C12:0	-2.19409	0	-0.71788	0	0.158774	0.11044	-1.2129	0	-0.30621	0.00876
C14:0	-1.31993	0	-1.26872	0	-0.60043	NA	-1.2344	0	-0.18727	0.0574
C14:1 n5	-0.58855	0	-0.93313	0	-1.82597	0	-1.0057	0	0.805776	0
C16:0	-0.60198	0.00258	-0.98017	0.00343	-1.72062	0.00081	-0.27204	0.02882	0.597363	0.00132
C16:1 n7	0.128662	0.06238	-0.85015	0	-2.42045	0	-0.84317	0	0.847156	0.00132
C18:0	0.015506	NA	-0.8457	0.00343	-0.86127	0.01789	-0.03925	NA	0.208886	NA
C18:1	0.195942	0.05793	-0.6794	0.0135	-1.35193	0.00219	-0.50146	0.00144	0.961968	0
C18:2 n6	-0.08994	NA	-0.62876	0.03334		NA		NA		NA
C18:3 n3 / C18:3 n6	0.682812	0.01531	-0.58787	0.02158		NA		NA		NA
C20:0	-0.61754	0.00258	-0.91554	0.00224	0.064478	NA	-0.57381	0.00144	-0.57899	0.01052
C20:1	0.325483	0.04783	-0.60542	0.04112	1.285774	0.03253	-0.69918	0.00052	-0.27814	0.03988
C22:0	-1.00158	0.01531	-0.77307	0.11073		NA		NA		NA
C22:1 n9	0.826484	0.0094	-0.70583	0.04112	1.811944	0.00496	-1.09237	0	-0.00393	NA
3-OH-3-Me-Glutaryl-CoA	-0.17946	NA	-0.82541	0	0.337164	0.01789	-0.70503	0	0.565832	0

Mevalonate	-3.40381	0	0.066299	0.27871	-1.89058	0	-0.65308	0	0.338768	0
Citrate	1.803074	0	-1.73512	0.00343	2.732578	0	0.02649	NA	0.468982	0
Aconitate		NA		NA	2.715891	0	-0.1349	NA	1.188727	0
Isocitrate	2.87182	0	-0.34672	0.21038		NA		NA		NA
alpha-Ketoglutarate	1.934468	0	-0.46472	0.0135	-0.17169	NA	-0.2188	0.00399	-0.50175	0.00132
Succinate	-3.46186	0	-0.22685	0.18075	-0.07041	NA	-0.84382	0	0.425706	0.00072
Fumarate	2.888445	0	-0.87362	0.02158	1.787291	0	-0.51684	0	-0.2791	0.02912
Malate	2.098385	0	-0.47974	0.00224	2.068362	0	-0.32331	0	-0.02469	NA

Supplementary References

1 Beyer, A. *et al.* Integrated assessment and prediction of transcription factor binding. *PLoS computational biology* **2**, e70, doi:10.1371/journal.pcbi.0020070 (2006).

2 Hibbs, M. A. *et al.* Exploring the functional landscape of gene expression: directed search of large microarray compendia. *Bioinformatics* **23**, 2692-2699, doi:10.1093/bioinformatics/btm403 (2007).
