# Supplementary material for: The Rad53CHK1/CHK2-Spt21NPAT and Tel1ATM axes couple glucose tolerance to histone dosage and subtelomeric silencing
Source: Nat Commun. 2020 Aug 19;11:4154. doi: 10.1038/s41467-020-17961-4 (PMC7438486; doi:10.1038/s41467-020-17961-4)
Supplement: Supplementary file 3 — Description of Additional Supplementary Files [file 41467_2020_17961_MOESM3_ESM.pdf]

Description of Additional Supplementary Files

File Name: Supplementary Data 1
Description: Gene expression analysis during carbon source switch
