# Supplementary material for: The Rad53CHK1/CHK2-Spt21NPAT and Tel1ATM axes couple glucose tolerance to histone dosage and subtelomeric silencing
Source: Nat Commun. 2020 Aug 19;11:4154. doi: 10.1038/s41467-020-17961-4 (PMC7438486; doi:10.1038/s41467-020-17961-4)
Supplement: Supplementary file 5 — Reporting Summary [file 41467_2020_17961_MOESM5_ESM.pdf]

nature research

Corresponding author(s): Christopher Bruhn
Marco Foiani

Last updated by author(s): Jul 10, 2020

Reporting Summary

Nature Research wishes to improve the reproducibility of the work that we publish. This form provides structure for consistency and transparency in reporting. For further information on Nature Research policies, see Authors & Referees and the Editorial Policy Checklist.

Statistics

For all statistical analyses, confirm that the following items are present in the figure legend, table legend, main text, or Methods section.

n/a	Confirmed	
☐	☒	The exact sample size (*n*) for each experimental group/condition, given as a discrete number and unit of measurement
☐	☒	A statement on whether measurements were taken from distinct samples or whether the same sample was measured repeatedly
☐	☒	The statistical test(s) used AND whether they are one- or two-sided<br>*Only common tests should be described solely by name; describe more complex techniques in the Methods section.*
☒	☐	A description of all covariates tested
☐	☒	A description of any assumptions or corrections, such as tests of normality and adjustment for multiple comparisons
☐	☒	A full description of the statistical parameters including central tendency (e.g. means) or other basic estimates (e.g. regression coefficient) AND variation (e.g. standard deviation) or associated estimates of uncertainty (e.g. confidence intervals)
☐	☒	For null hypothesis testing, the test statistic (e.g. *F*, *t*, *r*) with confidence intervals, effect sizes, degrees of freedom and *P* value noted<br>*Give P values as exact values whenever suitable.*
☒	☐	For Bayesian analysis, information on the choice of priors and Markov chain Monte Carlo settings
☒	☐	For hierarchical and complex designs, identification of the appropriate level for tests and full reporting of outcomes
☒	☐	Estimates of effect sizes (e.g. Cohen's *d*, Pearson's *r*), indicating how they were calculated

Our web collection on statistics for biologists contains articles on many of the points above.

Software and code

Policy information about availability of computer code

Data collection	Xcalibur 3.1.66.10 (Thermo Fisher Scientific) for phosphoproteomics<br>Xcalibur 2.2 SP 1.48 (Thermo Fisher Scientific) for metabolite analysis<br>NIS-Elements 4.60 (Nikon) for image acquisition<br>Andor Driver Pack 3 (Andor) for image acquisition
Data analysis	bedtools 2.24.0 Galaxy server https://usegalaxy.org/<br>Sailfish 0.7.6 Galaxy server https://usegalaxy.org/<br>RStudio 1.0.153 RStudio Team, 2016 https://www.rstudio.com/products/rstudio/download/<br>DESeq2 1.16.1 Love et al., 2014 https://bioconductor.org/packages/release/bioc/html/DESeq2.html<br>SPELL 2.0.3 Hibbs et al., 2007 https://spell.yeastgenome.org/<br>STRING 11.0 https://string-db.org/<br>Cytoscape 3.5.1 https://cytoscape.org/<br>Jalview 2.10.5 https://www.jalview.org/<br>Image J 1.51d https://imagej.nih.gov/ij/download.html<br>SORCERER 5.1.1 (Sage N Research, Inc.)

For manuscripts utilizing custom algorithms or software that are central to the research but not yet described in published literature, software must be made available to editors/reviewers. We strongly encourage code deposition in a community repository (e.g. GitHub). See the Nature Research guidelines for submitting code & software for further information.

Data

Policy information about availability of data

All manuscripts must include a data availability statement. This statement should provide the following information, where applicable:

- Accession codes, unique identifiers, or web links for publicly available datasets
- A list of figures that have associated raw data
- A description of any restrictions on data availability

Raw data for all Figures and Supplementary Figures are available at Mendeley Data [http://dx.doi.org/10.17632/r2bhxmvpxr.1].
RNA-Seq data for this submission are available on GEO (GSE137091) [https://www.ncbi.nlm.nih.gov/geo/query/acc.cgi?acc=GSE137091].
Phosphoproteomics data for this submission are on PRIDE (PXD020272).

Field-specific reporting

Please select the one below that is the best fit for your research. If you are not sure, read the appropriate sections before making your selection.

☒ Life sciences ☐ Behavioural & social sciences ☐ Ecological, evolutionary & environmental sciences

For a reference copy of the document with all sections, see nature.com/documents/nr-reporting-summary-flat.pdf

Life sciences study design

All studies must disclose on these points even when the disclosure is negative.

Sample size	When working with experimental repeats of the same biological samples (Western blots, qPCRs, toxicity assays) we selected 3 samples per group for minimal statistics requirements and repeated the experiment with additional samples if a trend but no significance was observed. If two independent biological samples were used, we performed two experimental repeats per independent sample. Supplementary Figures 1g and 2h represent an exception, where we performed two independent experiments and show data from one of these. For subtelomere imaging we selected at least 100 cells per condition to obtain biologically representative per cell data. For metabolomics studies we selected 5-6 samples per condition based on the recommendation of the service provider. For RNA-Seq analysis, the minimum sample size allowing statistical analysis by DESeq2 was chosen (2). Key genes were validated by qPCR for several genotypes along the paper. We have not applied any predictive statistics for sample size selection. However, for all numeric data we determined statistical significance with appropriate statistical tests, suggesting that, in case of significant differences, inclusion of additional samples was not required.
Data exclusions	No data were excluded from the analyses.
Replication	Replicates were organized as separate experimental runs to ensure reproducibility. We have not experienced cases of non-reproducible data in this study.<br>Where representative images are shown, we observed similar results in a total of three experimental repeats of the same clones (Figures 1c, 1i, 2f, 2g, 4e, 5b, 5c), two experimental repeats of two independent clones (Figures 1f, Supplementary Figures 1f), or two experimental repeats of the same clones (Supplementary Figures 1g, 2d).
Randomization	Each genotype or treatment was paired with the respective controls, and the same setting was repeated independently several times. This excludes the linkage of systematic errors to a specific genotype or treatment.
Blinding	RNA-Seq and metabolome analyses were outsourced to external service providers without providing a desription on the expected experimental outcomes. Therefore, both analyses are unbiased during data acquisition. All other experiments were performed with knowledge about the underlying genotypes and treatments.

Reporting for specific materials, systems and methods

We require information from authors about some types of materials, experimental systems and methods used in many studies. Here, indicate whether each material, system or method listed is relevant to your study. If you are not sure if a list item applies to your research, read the appropriate section before selecting a response.

Materials & experimental systems

n/a	Involved in the study	
☐	☒	Antibodies
☒	☐	Eukaryotic cell lines
☒	☐	Palaeontology
☒	☐	Animals and other organisms
☒	☐	Human research participants
☒	☐	Clinical data

Methods

n/a	Involved in the study	
☒	☐	ChIP-seq
☒	☐	Flow cytometry
☒	☐	MRI-based neuroimaging

Antibodies

Antibodies used

Mouse monoclonal anti Rad53 (clone EL7, dilution for western blot 1:5) In house (Fiorani et al., 2008)

Mouse monoclonal anti-acetyl-lysine (clone T52, dilution for western blot 1:10) In-house (Ronzoni et al., 2005)

Mouse monoclonal anti c-MYC (clone 9E10, dilution for western blot 1:2000) Santa Cruz Biotechnology Cat# sc-40, RRID:AB_627268

Rabbit anti-histone H3 (dilution for western blot 1:5000) EpiCypher Cat# 13-0001

Rabbit anti-histone H4 (dilution for western blot 1:4000) Abcam Cat# 7311

Mouse monoclonal anti-Pgk1 (dilution for western blot 1:10000) Novex Cat# 459250

Mouse monoclonal anti-Porin (clone 16G9E6BC4, dilution for western blot 1:1000), Abcam Cat# 110326

Rabbit anti-GFP (dilution for western blot 1:5000), Amsbio, Cat# TP401

Goat anti-mouse IgG (H + L)-HRP Conjugate (dilution for western blot 1:20000) Bio-Rad Cat# 1706516

Goat anti-rabbit IgG (H + L)-HRP Conjugate (dilution for western blot 1:20000) Bio-Rad Cat# 1706515

Validation

We have validated histone H3 antibodies with histone truncation mutants for western blot analysis to confirm that the recognized bands correspond to endogenous histones. We have confirmed for histone H3 and H4 antibodies that the detected bands are in the identical position as acetylated H3 and H4 bands.

Mouse monoclonal anti Rad53 (clone EL7) antibody has been used in various papers in S. cerevisiae for Western blotting (e.g. Ferrari et al., 2017, http://dx.doi.org/10.1016/j.molcel.2017.05.027)

Mouse monoclonal anti-acetyl-lysine (clone T52) antibody has been characterized for Western blotting in (Ronzoni et al., 2005, https://doi.org/10.1002/cyto.a.20151). The N-terminus of both H3 and H4 is highly conserved across species. In addition, we confirmed that the bands appear at the exact molecular weight positions.

Mouse monoclonal anti-Pgk1 antibody has been used in various papers in S. cerevisiae for Western blotting (e.g. Gay et al., 2018, https://doi.org/10.1016/j.molcel.2018.04.020). The product website specifically lists yeast as recognized species, and Western blotting as application.

Mouse monoclonal anti c-MYC antibody is commonly used and recognizes the MYC tag epitope in Western blot according to the product website. We also show the expected size of the tagged proteins.

Mouse monoclonal anti-Porin antibody: The product website specifically lists yeast as recognized species, and Western blotting as application. We also show the expected size of the protein.

Rabbit anti-GFP is commonly used and recognizes the GFP tag epitope in Western blot according to the product website. We also show the expected size of the tagged proteins.
